# The impact of solvent selection on the characteristics of niosome nanoparticles prepared by microfluidic mixing

**DOI:** 10.1016/j.ijpx.2023.100168

**Published:** 2023-02-04

**Authors:** Mohammad A. Obeid, Saja Haifawi, Ibrahim Khadra

**Affiliations:** a Department of Pharmaceutics and Pharmaceutical Technology, Faculty of Pharmacy, Yarmouk University, Irbid, Jordan; b Strathclyde Institute of Pharmacy and Biomedical Sciences, University of Strathclyde, 161 Cathedral Street, G4 0RE Glasgow, United Kingdom

**Keywords:** Niosomes, Organic solvent, Aqueous solvent, Microfluidic mixing

## Abstract

The aim of this work was to assess the impact of solvent selection on the characteristics of niosomes prepared by microfluidic mixing. To achieve this, niosomes were manufactured using bench-scale microfluidic mixing systems by changing the type of aqueous and/or organic solvents used to prepare the particles. Niosomes were prepared using different non-ionic surfactants and cholesterol compositions with different solvents and evaluated to investigate the influence of organic and aqueous solvents on the particle's physiochemical characteristics. Here we demonstrated that the solvent selection is a key factor to be considered during the preparation of niosomes with microfluidic mixing. The type of organic solvent was shown to significantly affect the size and the size distribution of the prepared particles. In general, niosome size increased with increasing organic solvent polarity, without affecting the niosomes stability. Moreover, changing the aqueous solvent used to hydrate the lipid components significantly (*p* < 0.05) affected the characteristics of the prepared niosomes in terms of particles size, size distribution, and surface charge. This impact of solvent selection on the final product is dependent on the lipid components where niosomes prepared with different compositions will have different characteristics when changing the type of organic and/or aqueous solvents. The apparent encapsulation efficiency of quinine as a model hydrophobic drug was subsequently shown to be significantly (*p* < 0.05) affected by the type of the organic solvent used to prepare the niosomes, while the impact of the organic solvent had less impact on the apparent encapsulation of atenolol as a model hydrophilic drug.

## Graphical abstract

**Figure:**
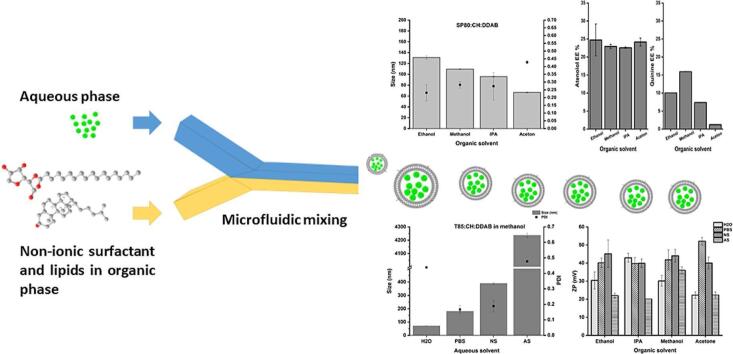

## 1 Introduction

Niosomes are a type of lipid-based carriers that are composed of non-ionic surfactants along with cholesterol and lipid charging agents. In the field of nanotechnology drug delivery, niosomes are considered versatile nanoparticles with the ability to encapsulate a range of hydrophilic and hydrophobic drugs such as anticancer agents, antifungal agents, and vaccines (Bhardwaj et al., 2020). Recently, there has been a rise in the use of niosomes as alternatives to liposomes for the delivery of already-approved drugs such as amphotericin B and doxorubicin in an attempt to improve their therapeutic effects and reduce their toxicity profile (Obeid et al., 2018; Alyamani et al., 2019).

Several methods have already been employed in niosomes preparation and most of them have been established for liposome preparation such as the thin film method, solvent injection method, heating method, and many others (Kaur and Kumar, 2018). However, most of the currently used methods for niosomes involve multi-steps and time-consuming procedures. Moreover, in many research studies, small and bench-scale methods are still being used, which have limited abilities to be translated into industrial manufacturing. This limits the possibility to translate these lab studies into a noisome-based product for clinical use (Webb et al., 2020; Obeid et al., 2022).

To overcome these limitations, a microfluidic mixing method has been investigated and employed recently for the preparation of both liposomes and niosomes. In the field of niosomes, the microfluidic mixing method offers a scale-independent process, which means easy transfer from bench to industrial scale production (Ag Seleci et al., 2019). This method for niosomes preparation involves the self-assembly of the lipid components, dissolved in an organic solvent, upon mixing with an aqueous buffer. The mixing process usually occurs under controlled mixing parameters such as the flow rate ratio (FRR) between the two phases and the total flow rates (TFR) (Maeki et al., 2017).

In previous reports, we have successfully demonstrated the use of microfluidic mixing for niosome preparation for the delivery of various small molecules (Obeid et al., 2020) and vaccines (Gebril et al., 2022).

Niosome production using microfluidic mixing involves the use of an organic solvent (normally alcohol such as ethanol or isopropyl alcohol, IPA) to dissolve the lipid components which is then mixed with the aqueous phase to promote the self-assembly of the lipids into a bilayer vesicular structure. The type of the organic solvent used must be able to dissolve the lipid components and must be miscible with water to promote the self-assembly of the dissolved lipids (Garcia-Salinas et al., 2018). Different organic solvents vary in their miscibility with water as a result of the differences in their carbon chain lengths and their surface tension. This solubility and miscibility of an organic solvent is related to the polar hydroxyl group and the carbon chain length, where an increase in the carbon chain length results in a decrease in the organic solvent polarity and solubility (Kinoshita et al., 1958).

Moreover, the solubility of any organic solvents in water is also governed by the level of hydrogen-bonding between the solvent and water. For example, ethanol is completely miscible with water because it has a short carbon chain length, and it can form hydrogen bonds with water molecules and with each other (Forster et al., 1991).

In the context of niosomes, the type of organic solvent used will not only affect the lipid solubility, but also has impact on the miscibility with the aqueous phase, which will eventually affect the self-assembly of the lipids into vesicular structures. Furthermore, the organic solvent must have a low toxicity profile even though it will be removed in the post-manufacturing purification steps (GUIDELINE, I. H. T., 2005).

Lipid assembly into vesicles has been described by Zook and Vreeland (2010) in which the self-assembly of the lipids into vesicles start with aggregation of lipids into discs where the hydrophobic chains around the edges are stabilised by the alcohol solvent. Upon decrease in the organic solvent concentration, these lipid discs start to bend and eventually close to form a spherical bilayer vesicle (Zook and Vreeland, 2010). Therefore, the solvent polarity is a crucial factor that will have impact on the initial lipid solubility as well as the process of vesicle formation. Among the available organic solvents for lipid nanoparticles preparation, IPA and ethanol are the most commonly used in the preparation of niosomes and liposomes using microfluidic mixing (Mijajlovic et al., 2013; Sangboonruang et al., 2021). However, very limited studies have explored the effects of the organic solvent on the characteristics of liposomes prepared by microfluidic mixing and to our knowledge no reports have investigated the effects of the organic solvent type on the characteristics of niosomes prepared by microfluidic mixing.

In previous work, we have reported that the type of aqueous media used to hydrate the lipids has significant impact on the characteristics of the prepared niosomes in terms of size, size distribution, surface charge, and particles stability (Obeid et al., 2017; Obeid et al., 2021). This present work aims to investigate the effect of changing the type of organic solvent and/or aqueous solvent during the microfluidic mixing process on the characteristics of niosomes in terms of their physicochemical parameters such as average particle size, polydispersity index (PDI), surface charge (zeta potential, ZP), stability, and drug encapsulation efficiency. For the latter one, atenolol and quinine were selected as a water soluble and bilayer soluble drugs respectively to evaluate drug encapsulation. To achieve this, both solvents have been changed and the prepared niosomes assessed accordingly.

## 2 Materials and methods

### 2.1 Materials

Tween 85 (T85), Span 80 (SP80), cholesterol (CH), didecyldimethylammonium bromide (DDAB), dicetyl phosphate (DCP), atenolol, quinine, phosphate buffered saline (PBS) (pH 7.4, 10 mM), normal saline (NS), ammonium sulphate buffer (AS) (pH 4.5), methanol, ethanol, acetone, and isopropanol (IPA) were purchased from Sigma-Aldrich (UK). All solvents and other chemicals were analytical grade.

### 2.2 Microfluidic production of niosomes

Cationic niosomes were prepared with T85:CH:DDAB and SP80:CH:DDAB at a 40:40:20 M ratio, while anionic niosomes were prepared with T85:CH:DCP and SP80:CH:DCP at a 50:40:10 M ratio. For microfluidic production of niosomes, each lipid component was dissolved in methanol, ethanol, acetone, or isopropanol to prepare stock solutions. The lipid phase of the formulations were prepared by mixing the required quantities to prepare the required molar ratios at an initial lipid concentration of 10 mg/ml. Niosomes were prepared by mixing each lipid phase with different aqueous phases through microfluidic chips obtained from Precision NanoSystems, Canada. The two phases were mixed through the microfluidic chips through the use of non-peristaltic syringe pumps obtained from VWR, USA. The aqueous media used to prepare the vesicles were either H_2_O, PBS (10 mM, pH 7.4), AS buffer (10 mM, pH 4.5), 0.9% (*w*/*v*) NS. The formulations were prepared at a FRR of 3:1 between the aqueous and lipid phase and the TFR was 8 ml/min. All formulations were prepared at 50 °C and the formulations after mixing were transferred to 15 ml Falcon tubes and further diluted with the same aqueous media used for the niosome preparation. For formulations prepared with atenolol, the drug was added to the aqueous phase so that the concentration was equal to 10% of the lipid concentrations after mixing. Whilst for the formulations prepared with quinine, the drug was added to the lipid stock solution at a concentration equal to 10% of the lipid concentrations (1 mg/ml).

### 2.3 Physiochemical evaluation of niosomes

Characterisation of particle size, PDI, and ZP were measured by dynamic light scattering using a Zetasizer Nano ZS (Malvern Instruments Ltd., UK). Zetasizer Software v.7.11 (Malvern Instruments Ltd.) was used for the acquisition of data. For the size, PDI, and ZP measurements, samples were diluted at a 1/10 dilution using the same aqueous phase used in the preparation of each one. i.e., samples were diluted either with 10 mM PBS (pH 7.4), H_2_O, 10 mM AS buffer (pH 4.5), or 0.9% (*w*/*v*) NS (pH 7.4) depending on the type of the aqueous media used in the preparation of each sample during the microfluidic mixing. The measurements were taken at 25 °C.

### 2.4 Niosome stability studies

Niosomes were stored at 4 °C and their size and PDI were measured over 5 days as above.

### 2.5 Removal of free drug with dialysis

Unencapsulated atenolol or quinine in the drug loaded formulations were removed by dialysis using dialysis tubing with a molecular weight cut-off 14 kDa. One ml of each formulation was dialysed against 500× of the same aqueous media used in the preparation of the formulation. The removed unencapsulated drug was measured in the dialysis media by UV measurement using JENWAY Genova Nano spectrophotometer and the dialysis was carried out until no more drug was detected in the dialysis media.

### 2.6 Characterisation of drug encapsulation efficiency (EE)

After removal of unencapsulated drug, the encapsulated drug contents (either atenolol as a model hydrophilic drug or quinine as a model hydrophobic drug) were determined using high performance liquid chromatography (HPLC) using an Agilent Technologies 1260 Series Liquid Chromatography system controlled by Clarity Chromatography software. For atenolol, the conditions of the run were: mobile phase PBS: methanol (70:30 *v*/v) pH: 6, flow rate 1 ml/min, total run time 8 min; column YMC basic C18, 250 × 3.0 mm, column temperature 40 °C, injection volume 20 μL, detection 275 nm, retention time 4.75 min. A standard curve of atenolol (39–2500 μg/ml) was constructed by measuring the area under the curve (AUC). Niosomes loaded with atenolol were lysed with methanol (100%) and then analysed by HPLC. The HPLC conditions for quinine analysis were: mobile phase ethanol: acetic acid: H_2_O (20:4:76 *v*/v/v pH, 2), flow rate 1 ml/min, total run time 8 min; column YMC basic C18, 250 × 3.0 mm, column temperature 40 °C, injection volume 20 μL, detection 254 nm, retention time 8 min. A standard curve of quinine (15–1000 μg/ml) was constructed by measuring the area under the curve (AUC). Atenolol and quinine concentration were determined by measuring the AUC and calculating the concentration using the equation generated from the standard curve of each drug. Atenolol and quinine apparent EE were calculated as a percentage of the initial concentration used.

### 2.7 Statistical analysis

All experiments were performed in triplicate and one way analysis of variance (ANOVA) was used to assess statistical significance. Tukey's multiple comparison test and *t*-test was performed for paired comparisons. The statistical analysis was performed using Minitab software version 19. A value of *p* < 0.05 was considered to be statistically significant. Graphs were produced using OriginPro 2021.

## 3 Results

### 3.1 Impact of the organic solvent on niosome physicochemical characteristics

**Fig. 1:**
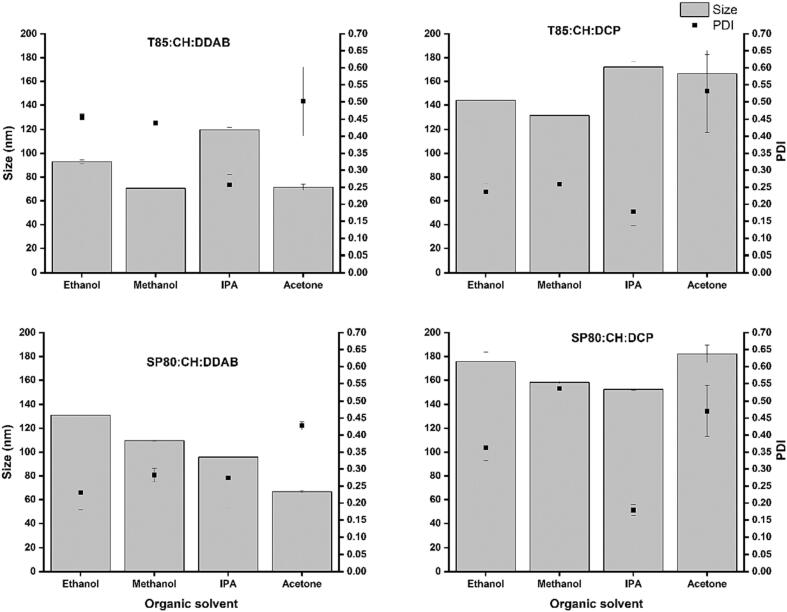
The impact of organic solvent on the particle size and PDI of different niosome formulations. Niosomes prepared by microfluidic mixing using different water miscible organic solvents (methanol, ethanol, acetone, or IPA) and H_2_O as the aqueous phase. Results represent the average ± SD of three measurements.

To investigate the effect of the solvent selection, a panel of niosome formulations were prepared by changing the organic solvent while fixing the aqueous solvent as ultra-pure water. Four different niosomes formulations were tested, which contained a combination of a non-ionic surfactant (85 or SP80), cholesterol, and charging lipid (DDAB or DCP for cationic and anionic niosomes, respectively). These formulations were tested first for their physiochemical properties in terms of size, PDI, and ZP. In Fig. 1A, niosomes prepared using T85, cholesterol, and DDAB, showed the particle size remained constant when using methanol and acetone (70.5 ± 0.6 nm and 71.4 ± 2.4 nm respectively). However, with ethanol and IPA the average size increased to 92.9 ± 1.6 nm and 119.8 ± 2 nm, respectively. Similarly, when niosomes were prepared with SP80, CH, and DDAB the average particle size decreased from 131 ± 3.0 to 66.7 ± 1.0 as the solvent changed from ethanol to methanol to IPA and to acetone (Fig. 1C). When the niosomes were prepared with SP80, CH, and DCP, the impact of the organic solvent was less as the differences in the particle sizes was small (Fig. 1D).

Regarding the effect of the organic solvent type on the dispersity of the nanoparticles, across all niosomes formulations, the PDI was in the range of 0.2 to 0.5, with methanol and ethanol having highest impact (Fig. 1).

**Fig. 2:**
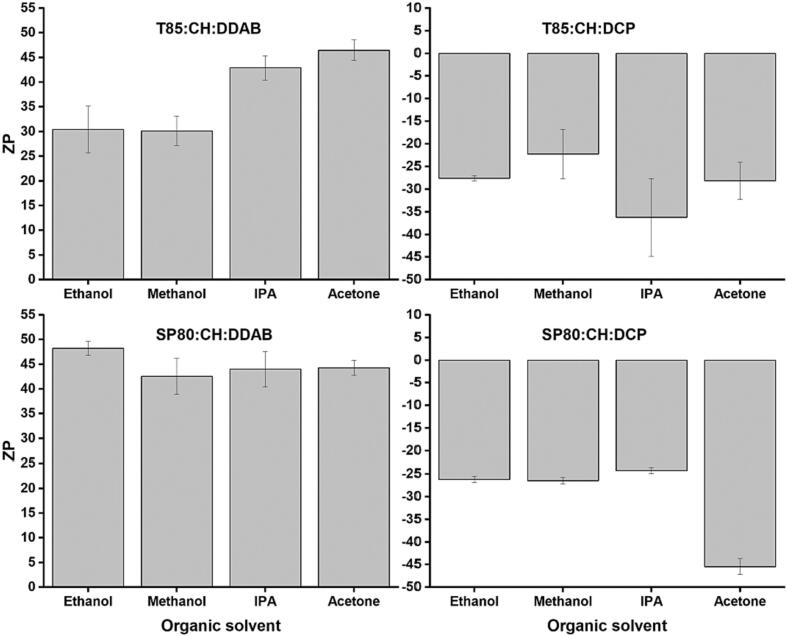
The impact of solvent selection on the ZP of different niosome formulations. Niosomes prepared by microfluidic mixing using different water miscible organic solvents (methanol, ethanol, acetone, or IPA) and H_2_O as the aqueous phase. Results represent the average ± SD of three measurements.

In terms of ZP values, two cationic and two anionic formulations were prepared with the incorporation of DDAB and DCP, respectively. Fig. 2 shows the effect of the organic solvents used on ZP.

### 3.2 Effect of organic solvent choice on niosome stability

The impact of organic solvent selection was also tested on the stability of the particles over five days by evaluating any changes in the size and PDI.

**Fig. 3:**
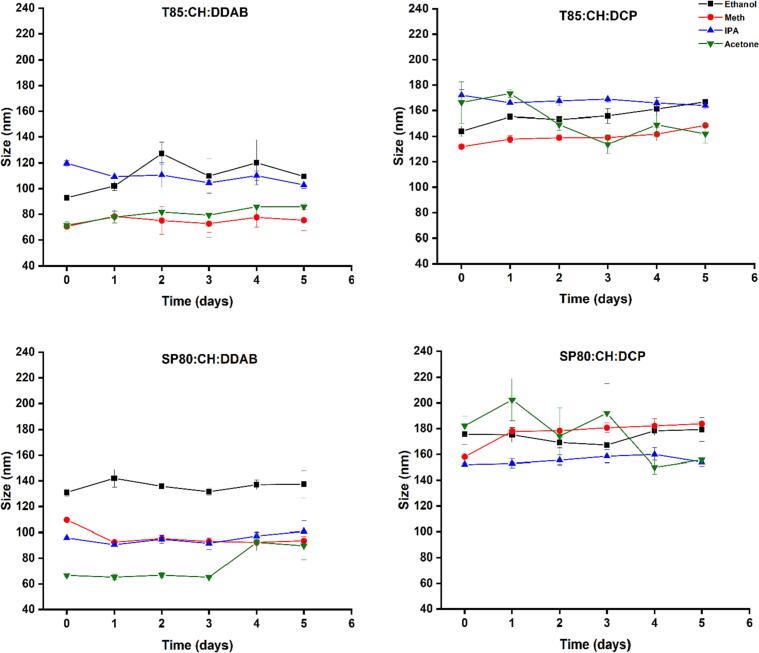
The stability of niosome nanoparticles over 5 days, in terms of size, prepared using different organic solvents. Results represent the average ± SD of three measurements.

**Fig. 4:**
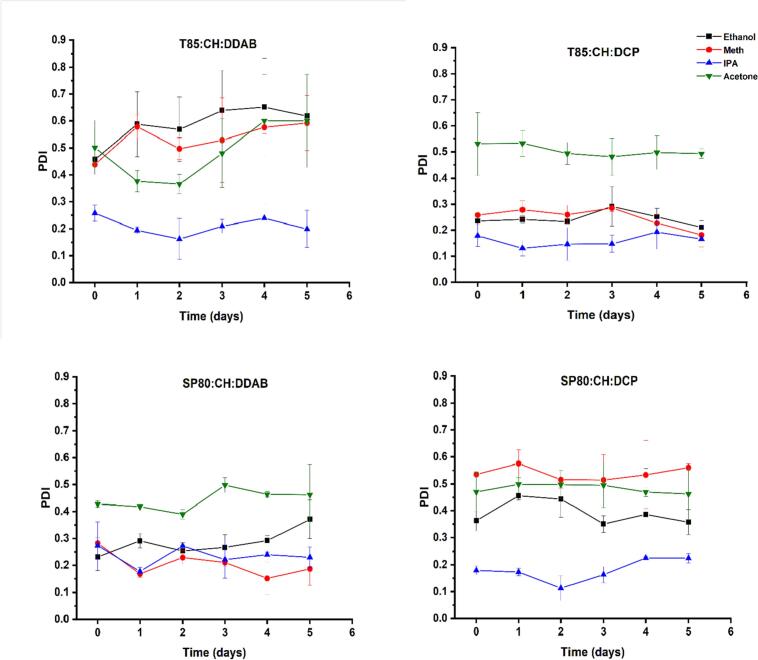
The stability of niosome nanoparticles over 5 days, in terms of PDI, prepared using different organic solvents. Results represent the average ± SD of three measurements.

Fig. 3 shows that despite differences in the particle size, PDI, and ZP, the formulations using ethanol, methanol, IPA all showed good stability whereas with acetone there was fluctuation and change of the particle size over time especially for niosomes prepared using DCP as the charging lipid. In terms of the dispersity of the particles, the PDI values indicate that the particles were stable over five days except for the first formulation prepared with acetone where the PDI values increased significantly (*p* < 0.05) over the study duration indicating the possibility of particle aggregation on storage (Fig. 4).

### 3.3 Organic solvent choice influences the drug encapsulation

**Fig. 5:**
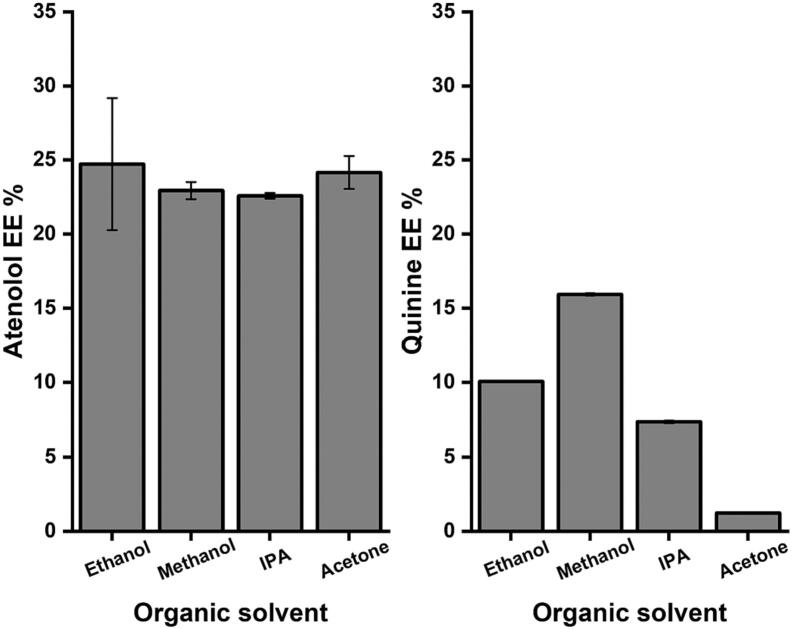
Apparent EE of atenolol in the aqueous core and quinine in the bilayer for niosomes prepared using microfluidic mixing. Niosomes were prepared with T85:Chol:DDAB and H_2_O as the aqueous phase. Results represent the average ± SD of three measurements.

The effect of changing the organic solvent in terms of atenolol and quinine loading as models for hydrophilic and hydrophobic drug, respectively are seen in Fig. 5 where the apparent EE values show that the type of the organic solvent significantly (*p* < 0.05) affects the level of the encapsulation of quinine at higher levels compared to atenolol.

### 3.4 The impact of changing the organic and the aqueous solvents together

**Fig. 6:**
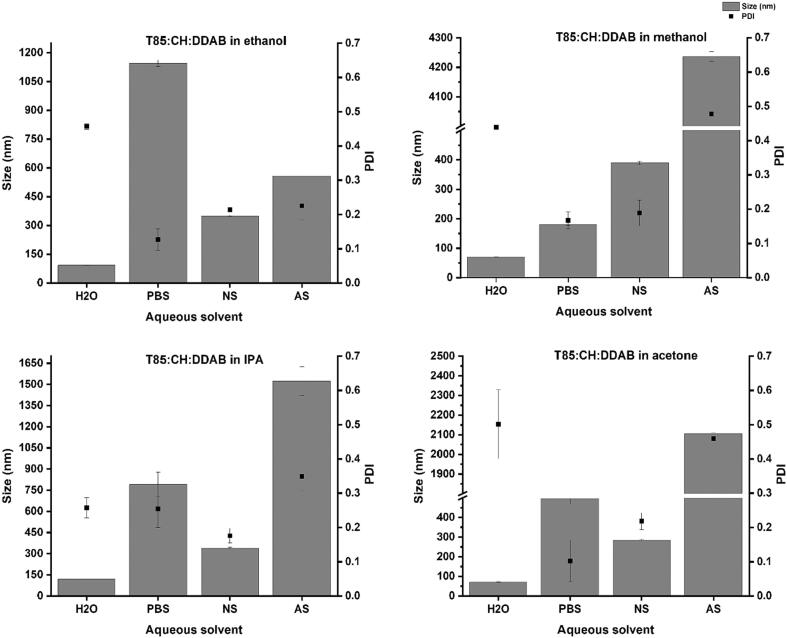
The effect of changing the aqueous and organic solvents on the size and PDI values of niosome nanoparticles. Results represent the average ± SD of three measurements.

**Fig. 7:**
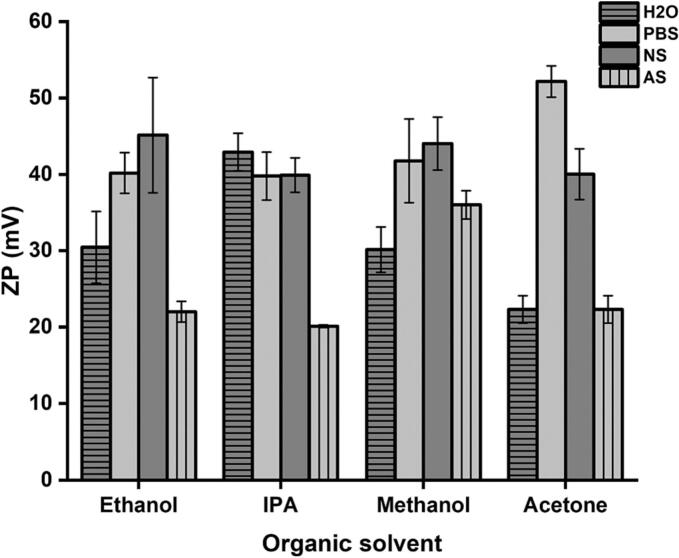
The effect of changing the aqueous and organic solvents on the ZP values of niosome nanoparticles. Results represent the average ± SD of three measurements.

Next, the effect of changing both the aqueous and organic solvents was evaluated. Fig. 6 represent the values of size and PDI and Fig. 7 the ZP values for these formulations.

These results indicate that the combinations of organic and aqueous phases significantly affect the particle characteristics. The use of H_2_O as an aqueous solvent resulted in niosomes with the smallest size among the other aqueous solvents, especially when methanol or acetone were used as the organic solvents. On the other hand, the use of AS as the aqueous solvent resulted in the preparation of large niosomes, especially when combined with methanol, acetone, or IPA. In terms of particle distribution, the PDI values were <0.5 in all the prepared formulations regardless of the type of aqueous/organic solvents combinations.

## 4 Discussion

Microfluidic mixing is an effective tool for the precise preparation of lipid nanoparticles such as liposomes and niosomes. This method can be easily scaled up for industrial production (Carugo et al., 2016; Zizzari et al., 2017). This potentially facilitates the translation of bench scale production into clinical products. With regards to niosomes which are prepared using non-ionic surfactants, several factors affect their application as drug delivery systems, including their size which eventually affects their biodistribution after administration, their surface charge which affects their rate of elimination and their stability.

The preparation and the control of the physiochemical characteristics of the produced particles can be affected by the design of the micromixer (Phapal and Sunthar, 2013), and the rates and ratios of the microfluidic mixing between the preparation phases (Zizzari et al., 2017; Forbes et al., 2019; Sangboonruang et al., 2021). These factors have been investigated and reported previously. However, the effect of the solvent selection has not been investigated in the literature extensively for niosomes, with a few reports highlighting these effects for liposome and niosomes preparations (Webb et al., 2019). We have reported previously that changing the hydration media or the aqueous phase significantly affects the niosomes characteristics (Obeid et al., 2017). In the present study, we investigated the effects of changing the organic and aqueous solvents. Across all the prepared formulations, the changes in particle size when switching the organic solvent between methanol and ethanol were generally low (Fig. 1).

During microfluidic mixing, the dilution of the organic solvent with the aqueous solvent will increase the medium polarity and forcing the intermediate disk-shaped lipid formed structures to self-assemble into a bilayer vesicles. Through this process, the formed particles might encapsulate remnants of the organic solvent. Therefore, the properties of the organic solvent might have effects on the characteristics of the prepared vesicles. The differences in the organic solvents polarities might be one of the reasones for these observed variations in the niosomes charactarestics. However, other factors might also affect the physichochemical properties of the nisomes prepared by microfluidic mixing such as the micromixer geometry, the ionic strength of the aqueous solvent, the micrfluidic mixng temprature, and the nanoparticles compositions and concentration (Damiati et al., 2018).

In this report, we have fixid the factors of the microfluidic mixing such as the temprature, the FRR, and the TFF to exclude their effects on the reported results. Moreover, the increase in the niosomes composition concentration were reported to result in the increase in the particles size (Aghaei and Solaimany Nazar, 2019). Therefore, to exclude the effects of the composition concentration of the reported results, this factor has been fixed and all the formulations were prepared at the same starting concentration.

Previous reports for changing the organic solvents for liposomes prepared with microfluidic mixing indicated the increase of the liposome size with decreasing organic solvent polarity (Webb et al., 2019). However, here with niosomes, the effect of solvent polarity was not apparent as no direct relationship was observed between the changes in the solvent polarity and the particle size. Methanol has the highest polarity of 0.762, followed by ethanol and IPA of 0.654 and 0.546, respectively while acetone has the lowest relative polarity of 0.355 (Kinoshita et al., 1958). In terms of particle size, there was no obvious changes with respect to the order of the relative polarity, which means the type of the organic solvent and not the polarity affects the size. These results show that we can control the particle size by controlling the type of organic solvent used. Similar results were reported by Zidan et al. (2017) who prepared niosomes while changing the organic solvent from ethanol to propylene glycol, to glycerol. This change of the organic solvents with this order was associated with a significant increase in particle size with a monodisperse distribution for all formulations (PDI <0.2) (Zidan et al., 2017).

Webb et al. (2019) explains the effects of the organic solvents on liposome characteristics by the effect of the organic solvent used on the formation of the discs during the process of the lipid self-assembly. They reported that the increase in polarity of the organic solvent used during the mixing process of the alcohol and buffer will result in the formation of the discs. While the reduction in polarity of the alcohol used (from methanol to IPA) will be accompanied by a reduction in the rate of change in polarity during the mixing process. This will eventually associate with the formation of larger discs and subsequently the formation of larger liposomes (Webb et al., 2019). Since niosomes have the same structure as liposomes with the only difference being the use of non-ionic surfactants instead of phospholipids, the same explanation could be applied to the results reported here especially for niosomes prepared with T85.

Moreover, Zook and Vreeland (2010) reported that the use of IPA provides additional stabilisation for the formed lipid discs since IPA has a long carbon chain length and this will contribute to the formation of larger particles compared to the use of other solvents (Zook and Vreeland, 2010). This is also notable in the present work where the use of IPA resulted in the formation of large niosomes especially when T85 was used as the non-ionic surfactant. Similar results were reported by Lopez et al. where they prepared liposomes with microfluidic mixing using different organic solvents and they found that the particles charactarestics depends on the type of the solvent used. They have attributed the differences in the particles charactarestics to the difference in the polarities of the organic solvent used for the preperation of the bilayer vesicles and the polarity change during the process of micromixing (López et al., 2021).

The use of organic solvent with low polartiy will be translated into higher polarity gradient upon mixing with the aqueous solvent in the micromixer. This icrease in the polarity gradient during the microfluidic mixing could also explain why the use of different organic solvent might result in niosomes with different characteristics. Another possible reason for the effect of the organic solvent on the nisomes properties is the concentration of the organic solvent used as the organic solvent is the concentration region in which niosomes are formed upon microfluidic mixing (Jahn et al., 2004).

Moreover, the differences in the organic solvent viscosities might have effects on the charactarestics of the prepared niosomes with microfluidic mixing. Different organic solvents have different viscosities. Previous work demonstrated a possible relation between the organic solvent viscosity and the mixing speed in the microchannel during the microfluidic mixng where the increase in the solvent viscosity would result in slower mixng during the fluids flow and this might result in the preperation of larger vesicles (Wu and Nguyen, 2005).

However, in our results, the effect of specific organic solvent on the particles characteristics was also dependent on the composition of the niosomes versicles and the type of the non-ionic surfactant. For example, although IPA has higher viscosity than ethanol and methanol at 20 °C, the particles prepared using IPA were sometimes smaller and sometimes larger depending on the niosomes composition (Fig. 1). This means that, similar to the solvent polarities, the organic solvent viscosities is not the only factor that control the particles properties and overall characteristics of niosomes depends on multiple factors. Previous studies reported counterbalanced effect of the organic solvents viscosities on the liposomes self-assembly process through the increase in the closure time and decreasing the growth rate (Zook and Vreeland, 2010).

As can be seen in Fig. 2A, for the cationic niosomes prepared with T85 as the non-ionic surfactant, methanol and ethanol resulted in particles with the same ZP while particles prepared with acetone and IPA had higher ZP values (42.9 ± 2.4 and 46.5 ± 2.1 respectively). However, methanol, IPA, and acetone resulted in the same cationic ZP for cationic niosomes prepared with SP80 and ethanol resulted in slightly higher ZP values (Fig. 2C). For anionic niosomes, no significant differences were seen across the anionic niosomes prepared using T85 when changing the organic solvent from ethanol to methanol to acetone, while IPA resulted in slightly more negative particles. For anionic niosomes prepared with SP80, only acetone resulted in a more negative ZP value which was significantly (*p* < 0.05) different to the ZP values when the niosomes prepared with other organic solvents. Across all the formulations, the particle size was also dependent on the type of the lipids used. For example, niosomes prepared with T85, CH, and DDAB using ethanol as the organic solvent had an average size of 92.9 ± 1.6 nm while using SP80 as a surfactant instead of T85 resulted in an average size of 131 ± 3 nm when prepared with ethanol (Fig. 1 A and C). This was also observed in all the other niosome formulations where the type of the surfactant and/or charging lipid affects the size of the resultant particles. This sensitivity of niosome composition on the organic solvent selection can be explained by the different characteristics of the non-ionic surfactants used and the different charging agents used such as the DDAB and the DCP.

Several studies have reported the effect of the alcohol used to change the bilayer free volume and hence affect particle characteristics (Ingólfsson and Andersen, 2011). Within the bilayer structure of liposomes or niosomes, the -OH groups of the alcohol will be positioned in the bilayer interfacial region while the hydrophobic methyl groups will be positioned in the hydrophobic core of the bilayer structure and disrupt the bilayer packing (Barry and Gawrisch, 1994). This explains why the type of alcohol and the lipids used will result in different particle characteristics (Ingólfsson and Andersen, 2011).

Despite these differences in size, PDI, and ZP, the niosomes formed using ethanol, methanol and IPA all showed good stability in terms of size and the type of organic solvent did not affect the stability of the prepared niosomes except for particles prepared with acetone where there was fluctuation in size over time especially for niosomes prepared using DCP. This can be explained by the fact that acetone has the least polarity among the other organic solvents. These results were the same as those reported for liposomes where ethanol, methanol, and IPA resulted in stable liposomes upon storage (Webb et al., 2019). In terms of the dispersity of the particles, the PDI values indicates that the particles were stable over five days except for the first formulation prepared with acetone where the PDI values increased significantly (*p* < 0.05) over the study duration (Fig. 4).

To further investigate the impact of changing the organic solvent during the microfluidic mixing, niosomes composed of T85: Chol: DDAB (40:40:20) and loaded with atenolol and quinine were prepared using different organic solvents and H_2_O as aqueous phase. When loading the niosomes with the hydrophilic drug atenolol, there was no significant difference in drug loading for niosomes when changing the type of the organic solvent. This can be explained by the fact that atenolol is water soluble and will be encapsulated in the aqueous moiety of the niosomes and hence the type of organic solvent does not affect its level of encapsulation. However, the loading of the hydrophobic drug quinine was significantly dependent on the type of organic solvent used. The use of methanol resulted in quinine apparent EE of around 15% while changing the methanol to ethanol and IPA reduced the level of quinine apparent EE to 10% and 7.5%, respectively. Acetone resulted in the preparation of niosomes with the lowest quinine apparent EE of <2.5% (Fig. 5). Here, the hydrophobic drug will be embedded in the bilayer structure and since the formation of this bilayer structure using microfluidic mixing was highly dependent on the type of the organic solvent then this can also explain these differences in the apparent EE of quinine by changing the organic solvent. The same results were reported by Zidan et al. (2017) for methotrexate into niosomes where they found that the solubility of methotrexate in the organic solvent employed in niosomes preparation significantly affected the apparent EE and this was explained by the variable distribution of methotrexate within the bilayer structure which is highly dependent on the type of organic solvent employed (Zidan et al., 2017). In the work of Webb et al. (2019) changing the organic solvent from methanol to ethanol did not significantly affect the liposomes apparent EE of the protein where the change was from 35% to 40%. However, the use of IPA in the same liposome preparations significantly (*p* < 0.05) lowered the protein apparent EE to 20% (Webb et al., 2019). Moreover, the effects of changing the niosome compositions and the type of non-ionic surfactant used on the characteristics of the prepared particles has been reported by many researchers. For example, Abdelbary and Aboughaly (2015) reported that changing the type of non-ionic surfactant significantly affect the size, PDI, ZP, and apparent EE of methotrexate in various niosomes formulations which was in agreement with the results reported in this work (Abdelbary and Aboughaly, 2015).

## 5 Conclusions

In this study, we have demonstrated that the type of solvent used in niosomes production using microfluidic mixing is considered among the key factors that should be considered. In this regard, changing the organic and/or the aqueous solvents will result in the preparation of niosomes of different particles size, size distribution, ZP, apparent EE, and stability. The effects of the aqueous solvents of the niosomes characteristics were reported to be due to the differences in the ionic strength and the salts concentrations (Obeid et al., 2017). This means that the solvent selection can be optimised to prepare niosomes with the required characteristics. In the early stages of niosomes formulation development using microfluidic mixing, different organic and aqueous solvents should be initially screened, and their effects should be fine-tuned. Therefore, niosomes size and all other characteristics are influenced by the type of alcohol used and the ionic strength of the aqueous buffer and both factors are considered as key factors in the development of niosomes using microfluidic mixing.

## Declaration of Competing Interest

The authors declare that they have no known competing financial interests or personal relationships that could have appeared to influence the work reported in this paper.

## Data Availability

Data will be made available on request.
